# Editorial: Quantitative wood anatomy to explore tree responses to global change

**DOI:** 10.3389/fpls.2022.998895

**Published:** 2022-09-09

**Authors:** Fabio Gennaretti, Marco Carrer, Ignacio García-González, Sergio Rossi, Georg von Arx

**Affiliations:** ^1^Forest Research Institute, Groupe de Recherche en Écologie de la MRC-Abitibi, Université du Québec en Abitibi-Témiscamingue, Amos, QC, Canada; ^2^TeSAF Department, Universitá degli Studi di Padova, Padova, Italy; ^3^Departamento de Botánica, Universidade de Santiago de Compostela, Lugo, Spain; ^4^Laboratoire sur les écosystèmes terrestres boréaux, Département des Sciences Fondamentales, Université du Québec à Chicoutimi, Chicoutimi, QC, Canada; ^5^Swiss Federal Institute for Forest, Snow and Landscape Research (WSL), Birmensdorf, Switzerland; ^6^Oeschger Centre for Climate Change Research, University of Bern, Bern, Switzerland

**Keywords:** quantitative wood anatomy (QWA), intra-annual resolution, tree functioning, trait plasticity, marginal populations, climate and environmental reconstructions, technical advancements

## Introduction

Quantitative wood anatomy (QWA) analyzes the anatomical traits of xylem and phloem cells in trees (von Arx et al., 2016). This research field is rapidly growing because anatomical traits may give in-depth insight into plant functioning and their dynamic responses to environmental change (Fonti et al., 2010). The size and structure of woody cells and their compartments are closely linked to tree hydraulic conductivity, drought resistance, and mechanical support (Tyree and Zimmermann, 2013; Hacke et al., 2015). Furthermore, QWA may offer information at a high temporal resolution over the growing season because each cell in the tree-rings is formed over a specific time frame and its anatomical traits may be dependent on the environmental constraints acting over this time frame (Puchi et al., 2020). The main drawback of QWA is the time and expertise needed to build anatomical time series. Most of the time QWA needs appropriate sample preparation techniques, microtomy, microscopy, and image analysis (von Arx et al., 2016). All these steps are time consuming and need special care to avoid systematic errors in the analyses.

This Research Topic aimed to gather a selection of relevant articles about QWA. Contributions are included showing how QWA can be used to understand the climate and environmental responses of trees at higher temporal resolution (intra-annual) than classical dendroecological studies. Other contributions use QWA to study tree functioning and their trait plasticity according to specimen provenance and growing site conditions, including marginal environments such as elevational treelines. Other contributions show the potential of QWA to improve climate and environmental reconstruction. Indeed, a likely future step forward in dendroclimatology will be to improve interpretations with a more in-depth understanding of tree functioning through QWA. Finally, the Research Topic includes some relevant technical advancements and standardized methods to improve the field of QWA in terms of reduction of analytic time, and improvement of reproducibility and interpretations.

## Intra-annual resolution to understand plant responses to extreme events

An increase in extreme climatic events is expected under global change and QWA may give special insights into the associated tree functional responses with intra-annual temporal resolution. In this context, Buttò et al. show that broad-leaved ring-porous and diffuse-porous species may have contrasted responses to drought, in some cases reducing the formation of additional xylem vessels and in others reducing their size. These changes are also simultaneous to shifts in canopy architecture. QWA may also be used to monitor wood formation processes (i.e., xylogenesis) in real time over the growing season with repetitive samplings each week. Gao et al. used xylogenetic monitoring to highlight the link between moisture availability and intra-annual density fluctuation in tree-rings of trees growing in arid and semi-arid regions of continental Asia with seasonal monsoon changes. Their findings are important to understanding possible plant responses to climate changes in this region.

## Tree functioning and their trait plasticity over environmental gradients

Another strength of QWA is that it potentially shows how trees are adapted or may acclimate (phenotypic plasticity) to different environments. Sampling European beech (*Fagus sylvatica* L.) populations differing in climatic regimes and spring leaf phenology, Arnič et al. show how mean vessel area, vessel density, and relative conductive area depend on precipitation and maximum temperature and may change over dry years. However, Miranda et al. highlight that European beech expresses limited anatomical trait plasticity and that simple interpretations may be distorted by the mixing effects of site conditions, land use changes, and ontogenetic patterns. Common garden experiments are needed to distinguish between plastic and genetic imprints on anatomical functional traits. For example, the study by Hietz et al. for the *Quercus robur* (L.) uses 10 tree provenances planted in three locations over a rainfall gradient to distinguish between the drivers of trait plasticity. Allometric dependences (ring width and tree size) accounted for stronger effects on all vessel parameters than site and provenance.

## QWA for studying marginal populations

Marginal populations are often studied in ecology because they are notably sensitive to environmental changes and may be used to understand potential biogeographical shifts with climate change. A better understanding of constraints and responses of tree species at the elevational/latitudinal treeline can be achieved by combining the classical dendroecological approach with QWA. This is the case in the study of Ştirbu et al. for *Pinus cembra* L. in the Carpathians where the authors combine tree-ring widths, density, and anatomical traits to assess the climatic sensitivity of those populations. The results show comparable correlations of maximum density and cell wall thickness with temperature and some negative impact of low water availability on conduit area. Similarly, Pampuch et al. sampled treeline populations of *Picea glauca* (Moench) Voss in Alaska showing that, among the anatomical traits, only cell wall thickness in the latewood is directly related to climate conditions, mainly temperature.

## Enhancing climate and environmental reconstructions

The direct links between environmental variability and anatomical traits may be used to improve climate and palaeoecological reconstructions with an enhanced mechanistic understanding of tree responses. In our Research Topic, three contributions explore these potential applications that are rapidly expanding in recent years. Balanzategui et al. show that lumen diameter of *Pseudotsuga menziesii* (Mirb.) Franco has a high potential to be used for temperature and precipitation reconstructions according to the analyzed sector in the tree-rings and such traits may be related to the decadal fluctuation of regional climatic modes (namely the Southern Oscillation Index). Tardif et al. explore the hydroclimatic sensitivity of flooded and non-flooded *Fraxinus nigra* (Marshall) trees concluding that only floodplain populations have strong and stable paleoclimatic signals in vessel anatomical chronologies. Using similar material, Nolin et al. reconstructed the local relationship between a river regulation by a dam and flooding episodes using flood rings. Such rings were noticeable by an increase in earlywood vessel number simultaneous to a decrease in vessel area.

## Technical advancements in QWA

New techniques and standard methods are highly sought in QWA because one of the main limiting factors of the discipline is the time and expertise needed to carry on the analyses. Thus, our Research Topic includes contributions describing new techniques that may potentially improve future analyses. A new workflow to study xylogenesis is proposed by Lehnebach et al. using high-resolution X-ray computed tomography. The most important technical advancement of this new technique is the possibility of precisely estimating biomass increments within the forming tree-rings for any tree species. This technique has the potential to provide relevant data to improve the modeling of carbon allocation and sequestration potential of forest stands (Gennaretti et al., 2017). Furthermore, Resente et al. show how an artificial intelligence algorithm may be trained to eventually save time and improve precision during the automatic detection of cell compartments. This artificial intelligence approach may be beneficial for future segmentation algorithms for QWA.

## Conclusion

The contributions to this Research Topic show several applications of QWA to better understand tree responses to global change and new technical advancements that will improve the discipline. QWA is rapidly expanding and evolving because it allows making the direct link between tree growth and tree functional responses. This collection of articles contributes to increasing interest in this discipline and identifying relevant future research directions.

## Author contributions

All authors acted as topic editors for this special issue in Frontiers in Plant Science entitled Quantitative Wood Anatomy to Explore Tree Responses to Global Change. All authors prepared the editorial text, sharing the tasks of writing, proofreading, and correcting the manuscript. FG initiated the idea of the special issue and organized the writing process of the editorial. All authors contributed to the article and approved the submitted version.

## Funding

This Research Topic and the collaboration between editors originated by projects funded by the Fonds de recherche du Québec - Nature et technologies (FRQNT) for the programme on the contribution of forest sector in mitigating the effects of climate change (grant no. 2022-0FC-309064). FG was supported by the Discovery Grants program of the Natural Sciences and Engineering Research Council of Canada (grant no. RGPIN-2021-03553). IG-G was supported by the Xunta de Galicia (grant no. GRC GI-1809). GA was supported by the Swiss National Science Foundation (grant no. 200021_182398, XELLCLIM).

## Conflict of interest

The authors declare that the research was conducted in the absence of any commercial or financial relationships that could be construed as a potential conflict of interest.

## Publisher's note

All claims expressed in this article are solely those of the authors and do not necessarily represent those of their affiliated organizations, or those of the publisher, the editors and the reviewers. Any product that may be evaluated in this article, or claim that may be made by its manufacturer, is not guaranteed or endorsed by the publisher.
